# Cohort of Patients Referred for Brugada Syndrome Investigation in an Electrophysiology Service – 19-Year Registry

**DOI:** 10.5935/abc.20180120

**Published:** 2018-07

**Authors:** Alvaro Valentim Lima Sarabanda

**Affiliations:** Instituto de Cardiologia do Distrito Federal, Fundação Universitária de Cardiologia (FUC), Brasília, DF – Brazil

**Keywords:** Brugada Syndrome, Bundle-Branch Block, Death, Sudden, Tachycardia, Ventricular, Syncope

Brugada syndrome (BrS) was described by Pedro and Josep Brugada in 1992 as a new clinical entity characterized by specific electrocardiographic (ECG) changes, such as the patterns of right bundle-branch block and persistent ST-segment elevation in right precordial leads, associated with increased risk for sudden death.^1^

Brugada syndrome is an autosomal dominant channelopathy, with clinical manifestation at the age of 30 to 40 years, affecting mainly men. Currently, BrS is estimated to account for 12% of all sudden cardiac deaths and up to 20% of the sudden cardiac deaths in individuals with no structural heart disease.^2^ The real prevalence of BrS in the general population is difficult to establish, being estimated at 5 to 20 in every 10,000 individuals.^2^ Several genetic mutations have been associated with BrS, most of them related to the encoding of sodium channel proteins (INa), calcium channel proteins (ICa) or potassium channel proteins (usually Ito) of the sarcoplasmatic membrane.^2-5^

The usual clinical manifestation of BrS is arrhythmic syncope, nocturnal agonal respiration or sudden death secondary to polymorphic ventricular tachycardia (VT) or ventricular fibrillation (VF). The symptoms usually occur during sleep, at rest during the day or in situations with increased vagal tone, such as binge drinking or heavy meals. In addition, fever is a common trigger, mainly among children.^2-5^

The diagnosis and risk stratification of BrS are mainly based on clinical history and ECG pattern, which can generate controversy because of the incomplete penetrance of the channelopathy and the dynamic pattern of ECG manifestations.^2-5^ Although three ECG patterns have been described, the diagnosis of BrS can only be established in patients with type 1 ECG pattern (coved-type), characterized by a concave ST-segment elevation ≥ 2 mm in at least two right precordial leads (V1, V2) positioned on the 2nd, 3rd and 4th intercostal spaces, occurring spontaneously or after provocative drug test with the intravenous administration of class I antiarrhythmic drugs (ajmaline, flecainide or procainamide). The other ECG patterns (types 2 and 3) do not establish the diagnosis of BrS.^2-5^

In BrS risk stratification and treatment, individuals with history of resuscitated cardiac arrest (class I indication) and those with history of syncope and type 1 ECG pattern (class IIa indication) are at high risk for sudden death and have indication for implantable cardioverter-defibrillator (ICD) placement for secondary prevention. Several studies have reported that asymptomatic patients with type 1 ECG pattern only after infusion of class I antiarrhythmic drugs are at low risk for arrhythmic events during clinical follow-up.^6-8^ Thus, in those patients, ICD placement should be avoided because of the risk of complications, such as inappropriate shocks.^3-5^

The role of programmed ventricular stimulation (PVS) during invasive electrophysiological study for risk stratification and management of asymptomatic patients with BrS is controversial. While some studies have shown that the induction of ventricular tachyarrhythmias (polymorphic VT or VF) during PVS is an independent predictor of arrhythmic events during clinical follow-up, emphasizing their negative predictive value,^9,10^ other studies have questioned those findings.^6,7^ It is worth noting the results of a recent systematic review of eight observational prospective studies, including 1,312 patients with BrS and no history of cardiac arrest, in which the induction of polymorphic VT or VF during PVS could predict an increased risk for arrhythmic events (cardiac arrest or ICD shocks) during clinical follow-up, with an increased risk for events when VT/VF induction occurred with only one or two extra-stimuli. However, those authors have reported that the lack of VT/VF induction could not predict a lower risk of arrhythmic events, especially in the subgroup of patients with type 1 ECG pattern and history of syncope.^8^ Thus, the most recent expert consensus recommend caution when indicating ICD placement in asymptomatic patients with BrS when ventricular tachyarrhythmias were induced by PVS, emphasizing the need to consider an individualized approach for ICD implantation (class IIb indication) in those patients.^3-5^

It is worth noting that, despite the description of BrS by the Brugada brothers more than 25 years ago,^1^ its genetic changes, arrhythmogenic mechanisms and clinical management continue to be debated. This is attributed to the continuous report of new information on BrS and its constantly evolving understanding, driven by new clinical and basic research findings.^2-8,10^

In the present *Arquivos Brasileiros de Cardiologia* issue, Warpechowski Neto et al.^11^ report the clinical characteristics, management and follow-up of patients with an ECG pattern suggestive of BrS, who had been referred to a tertiary center for risk stratification by use of invasive electrophysiological study. In the study, aligned with those previously reported in the literature, most patients were of the male gender, adults and had spontaneous type 1 ECG pattern. The study provides a timely and updated overview of the complexity of the BrS clinical management, discussing the controversies of using PVS for risk stratification of asymptomatic patients, as well as ICD placement to treat those at higher risk for fatal arrhythmias in the long-term clinical follow-up.
